# Eyewitness identification performance is not affected by time-of-day optimality

**DOI:** 10.1038/s41598-021-82628-z

**Published:** 2021-02-10

**Authors:** Sergii Yaremenko, Melanie Sauerland, Lorraine Hope

**Affiliations:** 1 Department of Clinical Psychological Science, Faculty of Psychology and Neuroscience, Maastricht University, PO Box 616, 6200 MD Maastricht, The Netherlands; 2 Department of Psychology, Faculty of Science, University of Portsmouth, Portsmouth, UK

**Keywords:** Psychology, Human behaviour

## Abstract

The circadian rhythm regulates arousal levels throughout the day and determines optimal periods for engaging in mental activities. Individuals differ in the time of day at which they reach their peak: Morning-type individuals are at their best in the morning and evening types perform better in the evening. Performance in recall and recognition of non-facial stimuli is generally superior at an individual’s circadian peak. In two studies (*N*s = 103 and 324), we tested the effect of time-of-testing optimality on eyewitness identification performance. Morning- and evening-type participants viewed stimulus films depicting staged crimes and made identification decisions from target-present and target-absent lineups either at their optimal or non-optimal time-of-day. We expected that participants would make more accurate identification decisions and that the confidence-accuracy and decision time-accuracy relationships would be stronger at optimal compared to non-optimal time of day. In Experiment 1, identification accuracy was unexpectedly superior at non-optimal compared to optimal time of day in target-present lineups. In Experiment 2, identification accuracy did not differ between the optimal and non-optimal time of day. Contrary to our expectations, confidence-accuracy relationship was generally stronger at non-optimal compared to optimal time of day. In line with our predictions, non-optimal testing eliminated decision-time-accuracy relationship in Experiment 1.

## Introduction

Life on earth revolves around a 24-h daylight cycle, and most organisms on our planet, including humans, function in compliance with this rhythm. This is possible by virtue of our internal body clock^1^. Research has demonstrated advantages to understanding how our internal body rhythms work and, whenever possible, making the timing of various activities congruent with this cycle^2–4^. Yet the effects of time-of-day cycles on eyewitness identification performance, which can play a crucial role in the administration of the law^5^, have received no attention in the research literature to date. We aimed to take the first steps in testing the possibility of time-of-day effects in these eyewitness identification contexts.

Our internal body clock generates the so-called *circadian rhythm* (from the Latin *circa*, meaning “around”, and *diem*, meaning “day”^6^) which ensures the proper timing of physiological and behavioural events. To maintain the optimum timing of rest and activity periods, the circadian clock regulates the level of physiological arousal throughout the day^7^. Not everyone’s circadian clock runs to exactly the same timings. Rather, people differ in their preferred time of the day for sleep and activity, as determined by their chronotype, or time-of-day preference^8,9^. Morning types, often referred to as “larks”, prefer waking up early and find it difficult to stay awake in the evening. Evening types, or “owls”, prefer to go to sleep late and have difficulties getting up early in the morning. Intermediate types show no strong morning or evening preference^10^.

Cognitive performance depends on whether or not the actual time of day is aligned with individual time-of-day preference^11^. Morning types reach their functional peak in the morning, whereas evening types are at their best in the evening hours. This phenomenon known as the *synchrony effect* has been shown to affect inhibition of distractors, non-relevant thoughts and unwanted responses^12,13^, automatic application of stereotypes and other judgmental heuristics^14^, implicit memory performance^15^, and accessibility of information from semantic memory^16^.

The synchrony effect is also observed in long-term memory performance. One study investigated whether matching or mismatching time of testing to participants’ time-of-day preference affected immediate recall of prose passages. Morning types recalled significantly more idea units at 9 AM (optimal) compared to the afternoon and evening sessions (non-optimal), whereas evening-type participants did not show a similar pattern of performance^17^. In another experiment, participants encoded a series of paragraph-length stories and then performed a verbatim sentence recognition task. The performance of evening participants improved from morning to afternoon, whereas performance of morning types was better in the morning, compared to the afternoon^18^. Non-optimal testing can also result in higher false memory rates compared to optimal testing in older but not younger adults and true recall and recognition of studied pictures but not verbal stimuli in both age groups^19^. These findings show that both recall and recognition memory are affected by time-of-day cycles in performance.

Synchrony effect patterns in long-term memory suggest that time-of-day optimality may be an important factor for eyewitness memory performance. Identification decisions can serve as important evidence in many cases, but can also lead to wrongful convictions and other forms of miscarriages of justice^5^. Are there reasons to expect that identification outcomes may vary as a function of the time of day when the crime was witnessed, or the lineup was administered? We tested this hypothesis across two experiments using an eyewitness identification paradigm. Participants watched a film depicting a staged crime and subsequently identified the individuals they saw in the film from lineups. Based on the previous findings showing superior long-term memory performance at optimal compared to non-optimal time of day, we hypothesized that participants would make more accurate identification decisions when testing time matched their diurnal preference.

We also investigated how chronotype synchrony affects the postdictive value of postdictors of identification accuracy, namely post-decision confidence and decision times. Typically, accurate choosers make their decisions with more confidence and faster than inaccurate choosers, whereas such associations between confidence, decision times and accuracy do not exist for nonchoosers^20–24^. There are reasons to expect that circadian asynchrony may have detrimental effect on the predictive value of confidence. For instance, confidence can be less predictive of accuracy in situations when encoding, retention and retrieval conditions are less optimal^25,26^. We tested the idea that chronotype asynchrony may be among such non-optimal conditions. We predicted that testing participants at their preferred times would strengthen the confidence-accuracy relationship in choosers. More specifically, we expected confident choosers to be more accurate than non-confident choosers and this relationship to be stronger at optimal compared to non-optimal time of day. We expected no such relationship for nonchoosers. Based on the findings that reaction times decrease across the day for evening types and increase for morning types^16^, we hypothesized that inaccurate choosers will take longer to make their decisions than accurate choosers, but even more so at non-optimal compared to optimal time of day.

## Experiment 1

### Method

#### Participants

We pre-screened 203 individuals who expressed interest in participation in the experiment for their circadian typology using the short form of Morningness-Eveningness Questionnaire (rMEQ)^27^. One-hundred-and-three pre-screened participants whose rMEQ score was 12 and lower (evening types) and 17 and above (morning types) were invited to participate in the main experiment (15 male, 87 female, 1 unspecified; age 18 to 58, *M* = 22.6, *Mdn* = 22 years). The sample consisted of university students (*n* = 98) and members of the general population (*n* = 4). About half of the sample consisted of evening-type (53.9%, *n* = 55, *M*_rMEQ_ = 9.82, *SD*_rMEQ_ = 1.88) and morning-type participants (46.1%, *n* = 47, *M*_rMEQ_ = 18.6, *SD*_rMEQ_ = 1.50). We recruited only Caucasian participants to avoid cross-racial bias in the identification task^28,29^.

#### Design

The experiment used a two-factorial mixed design with time-of-day optimality (optimal versus non-optimal) and target presence (absent versus present) serving as independent variables**.** Each participant was tested both at their optimal and non-optimal time of day. The order of optimality conditions (optimal–non-optimal vs non-optimal–optimal) was counterbalanced to control for potential learning effects. The order of stimulus films presentation was counterbalanced across optimality conditions.

Participants made a total of seven identification decisions across two testing sessions. Multiple-trial designs do not affect choosing rates and can be used to increase statistical power in eyewitness identification paradigms^30^. Target presence of each lineup was counterbalanced across the optimality conditions. Additionally, we partially counterbalanced the combinations of target presence conditions in each session: two of the lineups participants saw after encoding Film 1 were target-present and two were target-absent; similarly, after Film 2, participants received either one target-absent and two target-present lineups, or one target-present and two target-absent lineups. Identification accuracy (accurate versus inaccurate), post-decision confidence (on an 11-point scale ranging from 0 to 100%, with intervals marked in 10% steps) and decision times served as outcome variables.

#### Materials

##### Morningness-eveningness scales

We used the rMEQ^27^ to classify participants into morning- and evening-type categories. The rMEQ consists of five items drawn from the original full 19-item Morningness-Eveningness Questionnaire (MEQ)^8^. Both MEQ and its reduced version are commonly used to assess individual differences in diurnal preferences^10,31^. The use of the shorter scale allowed us to distract participants’ attention from the main hypothesis by combining the rMEQ items with filler questions about eating habits (e.g., “When you get up in the middle of the night, how often do you snack?”). The rMEQ score ranges between four and 25, with high scores referring to stronger morningness preference. We adopted cut-offs of ≤ 12 for evening types and ≥ 17 for morning types.

Additionally, we administered the full MEQ post-hoc following all the experimental manipulations as an extra validation of our classification of participants into chronotype groups. To establish test–retest reliability, we extracted participants’ responses to the five rMEQ items from a full version of the MEQ questionnaire that was administered at the end of the experiment. The results showed excellent test–retest reliability, *r*(100) = 0.92, *p* < .005.

##### Stimulus films

Two different stimulus films depicting the theft of a wallet were used. The films differ in the details of the event, the environment, and the actors. Film 1 depicts a theft taking place at a bar. Four amateur actors (1 female thief, 1 male victim, 2 female bystanders, 22–58 years old) appear in the film^32^. In stimulus film 2 the theft occurs in a university communal area. Three amateur actors (1 male thief, 1 female victim, 1 male bystander, 21–26 years old) appear in the film^33^.

##### Photo lineups

Six-person target-absent and target-present simultaneous lineups were constructed for each of seven targets (Film 1; one thief, one victim, two bystanders; Film 2: one thief, one victim, one bystander). Each lineup included six shoulder-up photographs that were arranged in two rows of three pictures and labelled 1 to 6. The target positions for the perpetrator, victim, bystander 1, and bystander 2 lineups in Film 1 were 4, 5, 4, and 3, respectively. The target positions for the perpetrator, victim, and bystander in Film 2 were 3, 6, and 4, respectively. The effective lineup sizes (Tredoux’s *E*s) were established in a pilot study, in which 19 to 38 mock witnesses (total *N* = 219) were presented with a description of the target and chose a lineup member that best matches the description^34^. Tredoux’s *E*s ranged from 3.6 to 5.6.

#### Procedure

##### Pre-screening

The protocol was approved by Ethics Review Committee Psychology and Neuroscience of Maastricht University. The experiment was performed in accordance with all the relevant guidelines and regulations. All participants provided informed consent to participate in the experiment. We told participants that the study concerned the effects of eating and caffeine-consumption habits on long-term memory in individuals with morning and evening time-of-day preference. Prospective participants who believed they had a morning or an evening preference contacted the research team, and were asked to fill out the pre-screening questionnaire to determine their eligibility to participate. The questionnaire consisted of five rMEQ items intermixed with filler questions concerning participants’ eating habits included to provide additional support for the cover story. Participants who fulfilled the pre-screening criteria were invited to participate in the main experiment. They were instructed to exclude alcohol or caffeine-containing products and sleep a minimum of 6 h prior to testing.

##### Main experiment

Data collection took place between March and June. Participants were tested individually on two separate occasions, which were scheduled in the morning (between 8:00 and 10:00) and in the evening (between 19:00 and 21:00). The two testing sessions took place with an interval of a minimum of 36 h (but no longer than a week) to avoid possible fatigue following a non-optimal session. The protocol for the two sessions was analogous, except where specifically indicated.

First, participants watched one of the two stimulus films. We instructed participants to pay close attention as they would later be asked to act as an eyewitness. Immediately after the film, participants provided a free narrative of what they remembered about the incident, including the sequence of actions and events, and described the appearance of the people involved. Next, participants answered 19 cued questions about the event (e.g., “Describe any interactions the thief/thieves has with the other people in the film”) and the appearance of the people involved in the incident, including their age, build, clothing etc.

Free reports and answers to cued questions were followed by a 30-min interval to avoid verbal overshadowing^35^. During this interval, participants filled in either the Pittsburgh Sleep Quality Index^36^ (Session 1) or demographic questionnaires (Session 2), followed by a visual version of DRM paradigm task (both sessions). The results of free and cued recall and the DRM tasks are reported elsewhere.

Next, participants identified individuals from the stimulus film. Prior to administering lineups, we presented a schematic of the crime scene with silhouettes of the individuals involved. The silhouettes were located in the same positions and had the same body postures as persons in the stimulus event (but provided no information about identity). Participants then viewed the lineups. The perpetrator lineup was always presented first. The remaining two or three lineups, respectively, appeared in a random order. Participants were informed which of the characters they will be asked to identify (“You will now view the lineup referring to the thief”). This instruction was accompanied by the aforementioned schematic of the crime scene in which the target character was highlighted in color, whereas the other individuals were displayed in grey. Participants were informed that the targets may or may not be present in the lineups and were encouraged to select the “Not present” option if they were not sure or didn’t know. Decision times for each identification decision were recorded automatically. After each decision, participants indicated their confidence.

At the end of the second session, participants filled out the full version of the MEQ. Participants received either gift vouchers worth € 27.50 or participation credit in return their participation and were debriefed via email upon the completion of data collection.

### Results and discussion

#### Data analyses

We used Generalized estimating equations (GEE) models to perform inferential analyses. GEE models are able to handle correlated data structures and thus can be employed to analyze binary outcomes collected from repeated measurements of the same individual^37^. The GEE model allowed us to include all seven measurements from each participant in the same model. An alpha level of 0.05 was used for all inferential analyses.

One participant showed an inconsistent classification according to rMEQ (the score of 12, i.e., an evening type) and the full version of the questionnaire, where he scored 57 (almost reaching the morningness cut-off of 59). Data for this participant were excluded from analyses.

#### Effect of time-of-day optimality on identification accuracy, choosing and other lineup outcomes

We conducted GEE analyses to establish the effect of time-of-day optimality (optimal versus non-optimal) and target presence (target-present versus target-absent) on identification accuracy (accurate versus inaccurate). We deleted non-significant terms stepwise. The final model included a significant Optimality × Target-Presence interaction, Wald χ^2^(1) = 3.93, *p* = .048. Simple slopes analyses revealed that participants made more accurate decisions at non-optimal compared to optimal time of day in target-present, Wald χ^2^(1) = 5.92, Exp(B) = 1.60, *p* = .015, but not target-absent lineups, Wald χ^2^(1) = 0.17, Exp(B) = 1.09, *p* = .680. That means, in target-present lineups, participants were 1.6 times more likely to make an accurate decision at non-optimal compared to optimal time of day. This finding was contrary to our expectations.

Following up on our unexpected findings, the GEE analyses were run in order to test whether lineup choosing rates, that is, rates of positive identifications as opposed to lineup rejections, were affected by testing optimality. Optimality (optimal versus non-optimal) and target-presence (target-present versus target-absent) were entered as predictors in the model; choosing (selection versus rejection) served as the outcome. The analysis revealed a significant Optimality × Target Presence interaction, Wald χ^2^(1) = 4.78, *p* = .029. Simple slopes analyses showed that participants were less likely to make a positive identification decision at optimal compared to non-optimal time of day in target-present, Wald χ^2^(1) = 14.21, Exp(B) = 1.91, *p* < .001, but not target-absent lineups, Wald χ^2^(1) = 0.06, Exp(B) = 1.05, *p* = .802. The odds ratio of 1.91 corresponds to a small effect size^38^. Table 1 displays frequencies and proportions of different lineup outcomes for each of the lineups. Lineup outcomes collapsed across the stimulus films can be found in Table 2.

**Table 1:** Frequencies and proportions of different lineup outcomes at optimal and non-optimal time of day in Experiment 1.

Lineup	Optimal		Non-optimal	
	Frequency	Proportion	Frequency	Proportion
**Film 1 Thief**				
*Target-present*				
Correct identifications	23	76.7%	30	81.1%
Foil identifications	4	13.3%	4	10.9%
Incorrect rejections	3	10.0%	3	8.0%
Total	30	100%	37	100%
*Target-absent*				
Correct rejections	10	55.6%	8	53.4%
Foil identifications	8	44.4%	7	46.6%
Total	18	100%	15	100%
**Film 1 Victim**				
*Target-present*				
Correct identifications	10	29.4%	7	21.2%
Foil identifications	8	23.6%	14	42.4%
Incorrect rejections	16	47.0%	12	36.4%
Total	34	100%	33	100%
*Target-absent*				
Correct rejections	9	64.3%	11	57.9%
Foil identifications	5	35.7%	8	42.1%
Total	14	100%	19	100%
**Film 1 Bystander A**				
*Target-present*				
Correct identifications	8	47.1%	22	64.7%
Foil identifications	0	0.0%	5	14.7%
Incorrect rejections	9	52.9%	7	20.6%
Total	17	100%	34	100%
*Target-absent*				
Correct rejections	17	54.8%	9	50.0%
Foil identifications	14	45.2%	9	50.0%
Total	31	100%	18	100%
**Film 1 Bystander B**				
*Target-present*				
Correct identifications	2	6.5%	2	11.0%
Foil identifications	7	22.5%	8	44.5%
Incorrect rejections	22	71.0%	8	44.5%
Total	31	100%	18	100%
*Target-absent*				
Correct rejections	11	64.7%	24	70.5%
Foil identifications	6	35.3%	10	29.5%
Total	17	100%	34	100%
**Film 2 Thief**				
*Target-present*				
Correct identifications	7	35.0%	14	45.1%
Foil identifications	2	10.0%	2	6.5%
Incorrect rejections	11	55.0%	15	48.4%
Total	20	100%	31	100%
*Target-absent*				
Correct rejections	21	67.7%	15	79.9%
Foil identifications	10	32.3%	4	21.1%
Total	31	100%	19	100%
**Film 2 Victim**				
*Target-present*				
Correct identifications	10	66.7%	24	70.6%
Foil identifications	1	6.6%	3	8.8%
Incorrect rejections	4	26.7%	7	20.6%
Total	15	100%	34	100%
*Target-absent*				
Correct rejections	26	76.5%	13	72.2%
Foil identifications	8	23.5%	5	27.8%
Total	34	100%	18	100%
**Film 2 Bystander**				
*Target-present*				
Correct identifications	18	53.0%	11	61.2%
Foil identifications	6	17.6%	5	27.7%
Incorrect rejections	10	29.4%	2	11.1%
Total	34	100%	18	100%
*Target-absent*				
Correct rejections	6	35.2%	13	40.6%
Foil identifications	11	66.8%	19	59.4%
Total	17	100%	32	100%

**Table 2:** Frequencies and proportions of different lineup outcomes collapsed across the stimulus films at optimal and non-optimal time of day in Experiment 1.

Lineup	Optimal		Non-optimal	
	Frequency	Proportion	Frequency	Proportion
*Target-present*				
Correct identifications	78	43.1%	110	53.7%
Foil identifications	28	15.5%	41	20.0%
Incorrect rejections	75	41.4%	54	26.3%
Total	181	100%	205	100%
*Target-absent*				
Correct rejections	100	61.7%	93	60.0%
Foil identifications	62	38.3%	62	40.0%
Total	162	100%	155	100%

Finally, we tested whether optimality affected choosing rates for targets as opposed to foils differentially. For this purpose, we conducted two GEE analyses with target and foil selections (selection versus no selection) as the outcome variables. There was a significant effect of optimality on target selection: Participants were more likely to select the target at non-optimal compared to optimal time of day, Wald χ^2^(1) = 5.75, Exp(B) = 1.58, *p* = .016, while the effect of optimality on foil selections was not statistically significant, *p* = .488.

##### Sensitivity and response bias

In light of the unusual way testing optimality affected choosing rates in target-present lineups, we conducted exploratory analyses drawing upon the signal detection theory approach^39^ to further clarify the observed effects. Signal detection theory is widely used in recognition literature to understand the mechanisms behind distinguishing novel items from those that were encountered before. For this purpose, signal detection analyses isolate hit and false alarm rates to compute two independent factors that affect recognition performance. *Discrimination accuracy* (*d′*) is a measure that can be used to distinguish signals (in identification task signal refers to responses to targets) from noise (responses to foils). A value of zero indicates zero ability to distinguish targets from non-targets. *Response bias* (*c*), on the other hand, refers to the threshold for deciding that participants have seen the target before. In the context of eyewitness identification, a negative *c* value indicates a bias towards making a selection from a lineup, whilst a positive *c* value shows a bias towards rejecting the lineup.

We tested the two *d′* and *c* values for optimal versus non-optimal conditions collapsed across all lineups. Discriminability was slightly, though non-significantly higher in non-optimal, *d′*_Non-Opt_ = 1.72, compared to optimal condition, *d′*_Opt_ = 1.34, *G* = 0.11, *p* = .915. Response bias was *c*_Non-Opt_ = 1.09 in the non-optimal sessions, compared to *c*_Opt_ = 0.94 in the optimal sessions. We are unaware of a significance test for the *c* values, but on a descriptive level, the values do not to show a substantial difference between response bias levels in the two experimental conditions.

**Figure 1:**
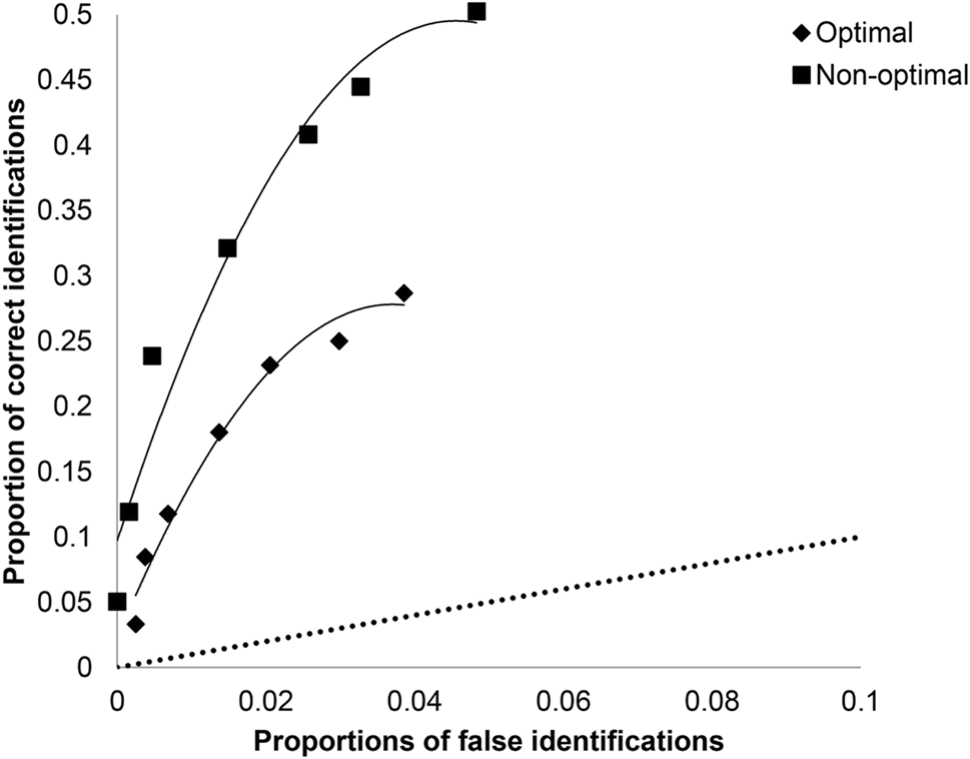
Receiver operating characteristics plots for optimal and non-optimal testing sessions across all lineups in Experiment 1.

Figure 1 displays the ROC curves for optimal versus non-optimal testing conditions. The curves were constructed by plotting cumulative hit rates against false alarm rates over decreasing confidence levels, with the diagonal line representing chance performance. Specifically, the *x* axis represents false alarm rates computed as the proportion of foil identifications in target-absent lineups divided by six (i.e., the lineup size)^40^, while the *y* axis represents the proportions of target selections in target-present lineups. Due to few observations in confidence levels 0 to 40%, we collapsed these categories. The comparison of the curves confirms results of the GEE analyses: the curves shows higher hit rates in non-optimal compared to optimal sessions at all the cut-off levels, whereas the false alarm rates were strikingly similar for the two conditions.

#### Effect of time-of-day optimality on the confidence-accuracy relationship

To establish the effect of time-of-day optimality on the confidence-accuracy relationship, we entered identification accuracy (accurate versus inaccurate), choosing (selection versus rejection), and time-of-day optimality (optimal versus non-optimal), as well as their two- and three-way interactions as predictors in the GEE model. Non-significant terms were deleted stepwise. The final model contained only the significant Identification Accuracy x Choosing interaction, Wald χ^2^(1) = 13.80, *p* < .001. Simple slopes analysis showed that accurate choosers were more confident than inaccurate choosers, Exp(B) = 3.09, Wald χ^2^(1) = 50.40, *p* < .001, whereas this was not the case for nonchoosers, Exp(B) = 1.22, Wald χ^2^(1) = 1.09, *p* = .297.

These findings replicate the common finding that accurate selections are made with more confidence than inaccurate selections^20–24^. However, contrary to our expectations, this interaction effect was not moderated by testing optimality.

##### Calibration of confidence measures

We conducted explorative calibration analyses, which can be an informative way to explore on the association between the objective accuracy probabilities and subjective post-decision confidence measurements. There are several ways to assess how well eyewitnesses are calibrated in their confidence judgements. First, the proportion of accurate decisions for each confidence level can be plotted against the mean confidence for the respective level to create a calibration curve. Visual inspection of the curve allows to assess how well-calibrated participants were in each of the levels of confidence. Second, the calibration statistic (C) provides a quantitative reflection of the level of deviation from perfect calibration. It ranges from 0 (perfect calibration) to 1 (poorest calibration). Third, the over/underconfidence (O/U) statistic is a further indicator of how well-calibrated participants are. It varies from − 1 to + 1, with negative scores reflecting underconfidence and positive scores showing overconfidence. Finally, the normalized resolution index (*NRI*) allows to evaluate how good participant’s confidence judgments are at discriminating accurate from inaccurate decisions. It ranges from 0 (lowest resolution possible) to 1 (perfect discrimination)^41^.

As expected and confirming the GEE analyses, the confidence-accuracy correlation was significant for choosers, *r*(380) = 0.36, *p* < .001, but not for nonchoosers, *r*(319) = 0.04, *p* = .478. Note the (nearly) large effect size for choosers that corresponds well with the effect reported in the meta-analysis^24^. Following Flowe et al., we collapsed the confidence categories into three categories (i.e., 0–40%; 50–70%; 80–100%) to provide more stable estimates for each confidence category^42^. The proportion of accurate decisions for each of the collapsed categories was plotted against the weighted mean confidence for that category to create the confidence-accuracy calibration curve.

**Figure 2:**
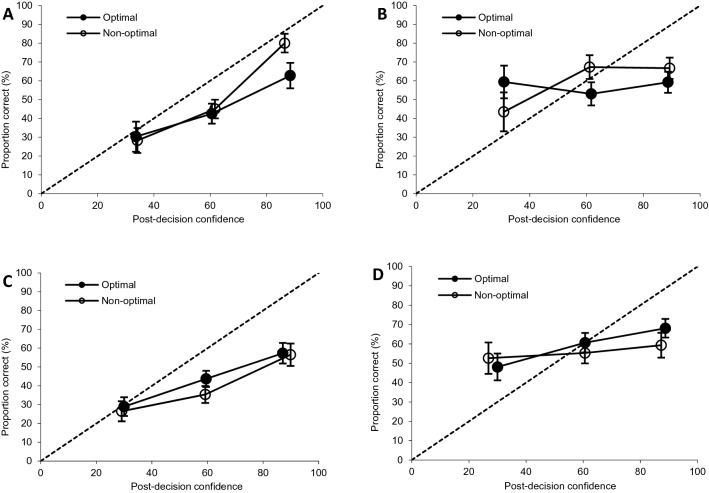
Post-decision confidence-identification accuracy calibration curves (and 95% CI bars) for optimal and non-optimal time-of-day in choosers (panel **A**) and nonchoosers (panel **B**) in Experiment 1 and for choosers (panel **C**) and nonchoosers (panel **D**) in Experiment 2.

Figure 2 (panel A) displays the confidence-accuracy calibration curves for choosers at optimal (number of observations *n* = 171) and non-optimal (*n* = 211) time of day. The comparison of confidence intervals reveals that calibration in the last category (80–100%) was significantly better at non-optimal compared to optimal time of day. Figure 2 (Panel B) shows the confidence-accuracy calibration curves for nonchoosers at optimal (*N* = 174) and non-optimal (*N* = 147) time of day. Similarly to choosers, the comparison of confidence intervals shows that calibration in one of the confidence categories, namely the second category (40–70%), significantly differed at non-optimal as compared to optimal time of day. Confidence intervals for the other confidence categories in choosers and nonchoosers overlap. Calibration statistics presented in Table 3 also show no significant differences between the optimality conditions.

**Table 3:** Calibration measures for optimal and non-optimal testing sessions split by choosers and nonchoosers in Experiments 1 and 2.

	Experiment 1				Experiment 2			
	Optimal		Non-Optimal		Optimal		Non-Optimal	
Choosers								
*C and 95% CI*	.036	.009; .064	.015	− .001; .032	.013	.000; .026	.023	.004; .041
*O/U and 95% CI*	.175	.104; .246	.112	.050; .174	.059	.003; .116	.116	.055; .176
*NRI and 95% CI*	.056	− .015; .126	.156	.061; .250	.066	.003; .130	.102	.022; .181
Nonchoosers								
*C and 95% CI*	.056	.020; .092	.028	.002; .054	.031	.008; .054	.031	.005; .057
*O/U and 95% CI*	.109	.027; .191	.063	− .019; .146	− .058	− .124; .007	− .008	− .086; .069
*NRI and 95% CI*	.037	− .034; .108	.004	− .015; .023	.019	− .017; .055	.004	− .014; .022

Confirming GEE analysis, these findings show no support for our hypothesis that postdictive value of confidence would be stronger at optimal time of day. In fact, calibration analyses show some evidence in the opposite direction: participants were better calibrated in some of the confidence categories in non-optimal compared to optimal sessions.

#### Effect of time-of-day optimality on the decision time–accuracy relationship

The decision time distributions showed significant positive skewness and kurtosis. Therefore, log-transformed decision times (log base 10). To establish the effect of time-of-day optimality on the decision-time-accuracy relationship, we entered identification accuracy (accurate versus inaccurate), choosing (selection versus rejection), and time-of-day optimality (optimal versus non-optimal) and their interactions in the initial GEE model. Log-transformed decision times served as dependent variable. The model revealed a significant three-way interaction, Wald χ^2^(1) = 4.03, *p* = .045. Simple analyses of the Accuracy × Choosing interaction revealed a significant interaction effect at optimal, Wald χ^2^(1) = 6.19, *p* = .013, but not at non-optimal time-of-day, Wald χ^2^(1) < 0.01, *p* = .954. Further analyses were performed on choosers and nonchoosers that were tested at optimal time-of-day. The simple simple effect of accuracy was significant neither for choosers, *b* = 0.07, Wald χ^2^(1) = 3.31, *p* = .069, nor for nonchoosers, *b* = − 0.07, Wald χ^2^(1) = 3.60, *p* = .058. These results confirm our hypothesis, showing that non-optimal testing eliminated the postdictive value of decision times. However, the simple simple effect analyses fail to show a common finding that accurate chooser decisions are faster than inaccurate chooser decisions^20–24^.

Overall, the findings of Experiment 1 are contrary to the literature on the effects of circadian rhythms on cognitive performance. Commonly, performance is superior at optimal compared to non-optimal time-of-day^13,18,19^. It is possible that despite the use of the cover story, participants were aware of the optimality manipulation due to repeated testing in the morning and evening hours. This may have resulted in more careful stimulus encoding or other strategies aimed at compensating for the anticipated cognitive impairments in the non-optimal session. It is however unclear why the change in target selections was not accompanied by an effect in false alarms, as it is commonly observed in recognition memory performance studies^43^. Combined with the small size of the observed effect, the need for replication is evident.

## Experiment 2

To further investigate the effect of time-of-day optimality on eyewitness identification performance, we conducted a second experiment. In this experiment, we recruited participants via Amazon MTurk, which allowed us to collect a more demographically diverse sample with increased statistical power. Additionally, we aimed to take a further step by introducing a memory bias in some of the experimental conditions. We were interested in testing the hypothesis that, if present in the lineup administration procedure, factors that are known to bias eyewitness’s memory would be magnified by non-optimal testing. As means of introducing bias, we attempted to use the so-called mug shot exposure effect. This effect describes the phenomenon that exposing eyewitnesses to mug shots before viewing a lineup may bias their decision^44^. More specifically, eyewitnesses may base their decision on familiarity gained due to the mug shot presentation rather than on their memory from the actual event, thus increasing the likelihood of innocent suspect misidentifications. Previous research showed that such automatic manifestations of memory can be magnified at non-optimal compared to optimal time of day^15^. We hypothesised that such erroneous identifications due to mug shot exposure would be more likely to occur during circadian troughs compared to circadian peaks.

### Methods

The experiment was pre-registered on the Open Science Framework. The registration form can be accessed at https://osf.io/kafe7.

#### Sample size

Power analysis for a two-tailed binomial logistic regression with G*Power v3.1 returned a required sample size of 310^45,46^. To achieve equal distribution of participants across the four experimental conditions, we tested two extra participants, which resulted in the planned sample size of 312. We used the following parameters: OR = 2.058; Pr(Y = 1|X = 1); H0 = 0.383; R^2^ other X = 0.2, X distribution = Binomial, X parm π = 0.5. We based the odds ratio on the corrected recognition scores reported in a previous study by May et al. showing the synchrony effect on recognition performance^18^. An alpha error probability of .05 and a power of .80 were used.

#### Participant pool and inclusion criteria

Participants were recruited with the help of Amazon Mechanical Turk (MTurk) platform. MTurk is an online crowd sourcing system that was developed with an aim to connect potential workers (“MTurkers”) with the so-called requesters, who offer jobs or tasks that can be completed online. Researchers have been increasingly using the MTurk platform for the purposes of data collection due to the multiple advantages it offers for academic research^47,48^. MTurkers do not appear to be significant outliers in terms general demographics, and in some aspects the platform offers access to samples that are more representative compared to student samples^48,49^. The psychometric properties of responses collected on MTurk have been validated, and multiple laboratories managed to replicate some of the classical findings using the platform^50,51^. Amazon MTurk offers opportunities for efficient and less costly data collection with reliable results. Combined with the fact that MTurkers are known to complete tasks around the clock, this has guided us in our decision to collect the data for Experiment 2 using the platform.

#### Data validity checks

Online testing can increase the rates of careless or partially random responses^52^, an issue which may be of extra concern in studies that rely on Amazon MTurk platform in participant recruitment^53^. Therefore, we took a series of additional measures to exclude random or careless responses and other problems related to the lack of experimental control in the MTurk environment.

##### Pre-screening

We included data quality check items in the pre-screening questionnaire, which allowed us to identify attempts of careless or automatic responses. One of the items paraphrased an MEQ question (”How difficult do you find it to get up in the morning (when you are not awakened unexpectedly)?” in the original version as opposed to “How easy do you find it to get up…” in the modified item). The other item duplicated an MEQ question (”At approximately what time-of-day do you usually feel your best?”) in a form of a text entry question, i.e., participants were required to manually type in the time-of-day in AM or PM format. Each of the questions of the pre-screening survey was presented on a separate page, and participants could not go back to check their previous responses. Second, we relied on the duration of the response to the pre-screening questionnaire as an indicator for random or careless responses. We considered response duration below 2 min and 30 s to be an indicator or careless or random responding for a survey containing 37 items. MTurkers who produced incorrect responses to the validity items or short response duration were not invited for participation.

##### Main experiment

Further data quality check items were included in the main part of the study. First, one of the MEQ items from the pre-screening questionnaire and three demographics questions were duplicated in the main experiment, allowing us to check for consistency of participant’s response to these items across the two parts of the study. Second, we included the Instructional Manipulation Check (IMC)^54^ in the concluding phase of the main part of the study. The IMC includes a question in a form of a block of text with lure responses, overall mimicking a typical multiple choice question. However, the long text block contains the instruction to submit a counterintuitive response. The accurate response to IMC could serve as one of the indicators that participants have been following the study instructions carefully. Finally, at the end of the experiment, participants had to answer two simple questions related to the content of the stimulus film. Specifically, participants were asked about the item that was stolen in the incident (multiple choice) and were instructed to indicate what the thief did with the stolen item (free entry field). Thus, one of the control questions referred to the middle of the stimulus film, while the other one concerned the detail at the end of the film. This allowed us to detect careless or inattentive MTurkers and was also helpful in identifying cases when participants did not encode the stimulus fully due to some technical problems. Participant’s response was counted as reliable only if they passed the IMC, showed consistent responses to the control items and responded to the questions related to the stimulus film accurately.

Due to the fact that participants on MTurk participated across several time zones, we used the location data to verify participants’ adherence to the requirements on participating within the specified timeframes.

##### Possible character misrepresentation

Another potential problem is the possibility of character misrepresentation in Amazon MTurk samples. More specifically, experiments that target specific populations may encourage some MTurkers to falsify their identities (i.e., claim to belong to certain categories of population) or make multiple attempts to pass the screener questions to qualify for the main study and receive higher reimbursement. To address this concern, we adopted a two-step pre-screening procedure to prevent MTurkers from falsifying the answers to the pre-screening questionnaire to qualify for the main experiment^55^. Specifically, we aimed to create an impression that the pre-screening questionnaire was actually an independent survey focusing on the way sleep habits affect eating and caffeine consumption behavior. There was no indication of a link between the pre-screening and the main study. The main study was visible only to MTurkers who were eligible and passed the initial data quality checks.

#### Participants

We continued data collection until we achieved the planned number of reliable responses. For this purpose, we pre-screened a total of 4,270 MTurkers. Among the pre-screened participants, 1,478 were morning-types and 568 were evening-types. A total of 363 proceeded to participate in the main study, of which 39 were excluded because they did not meet some of the data quality checks.

Upon completion of data collection, we discovered that the question asking participants to indicate their lineup decision appeared on a separate rather than the same page as the lineup itself for 12 of the participants. In the event that this formatting discrepancy produced any distortion in responses provided by these participants, we collected data for a further 12 participants.

Hence, the final sample consisted of 324 participants (160 male, 163 female, 1 unspecified; age 19 to 66, *M* = 35.6, *Mdn* = 34 years). A total of 118 of them showed evening preference (36.4%, *M*_MEQ_ = 35.8, *SD*_MEQ_ = 3.99), and 206 participants were morning types (63.6%, *M*_rMEQ_ = 64.03, *SD*_rMEQ_ = 4.59). Participants received a $3 honorarium on completion of the experiment.

#### Design

The study used a two-factorial mixed design with time-of-day optimality (optimal versus non-optimal) and target presence (present versus absent) as independent variables. Participants were randomly assigned to be tested either at their optimal or non-optimal time of day. We counterbalanced target presence of each lineup across the optimality conditions. Additionally, we partially counterbalanced the combinations of target presence in the three lineups: participants received either one target-present and two target-absent lineups, or one target-absent and two target-present lineups.

In the thief lineups, we additionally manipulated mug shot exposure bias (bias versus no bias). In the biased condition, one individual appeared among both the mugshots and the lineup foils. Only one participant in the biased conditions selected the innocent suspect from the lineup, suggesting that the mug shot manipulation did not bias lineup decisions. Therefore, this factor will not be discussed further.

Participants made three identification decisions. For each of the three lineups, target-presence was counterbalanced across the two optimality conditions. Identification accuracy (accurate versus inaccurate), post-decision confidence (on an 11-point scale ranging from 0 to 100%) and decision times served as dependent variables.

#### Materials

##### The pre-screening questionnaire

We used the full version of MEQ^8^ to identify participants’ time-of-day preference. The 19 original MEQ items were intermixed with filler questions related to participants’ sleep, food and caffeine consumption habits to mask the aim of the pre-screening survey and provide additional support for the cover story. Example items are “How much control do you have over your eating between supper and bedtime?”, “At what time do you prefer to drink your first caffeine-containing beverage?”, “When you get up in the middle of the night, how often do you snack?” Additionally, the questionnaire included the two data quality check items.

##### Stimulus film

We used Film 2 from Experiment 1 as the stimulus event.

##### Mug books

We constructed two mug books, containing 16 head-and-shoulder photographs of white males that met the description of the thief from the stimulus film. The biased mug book contained a photograph of the innocent suspect; this person would also appear in the thief lineup as the incidental target. The unbiased mug book included a photograph of a control foil.

##### Photo lineups

We created six-person target-present and target-absent simultaneous lineups for each of the three targets appearing in the stimulus film, namely, the thief, the victim and the bystander. Target-absent lineups were constructed containing photographs of fillers that matched the description of the target in a similar way as in Experiment 1. For target-present lineups, we created six versions of lineups, with all possible combinations of the target with five of the foils from the target-present lineup. The target positions in the thief, victim, and bystander lineup were 3, 6, and 3, respectively. The incidental target (i.e., the innocent suspect who appeared among the biased mug shots) took position 5 in the thief lineup. Additionally, we created six extra target-present thief lineups in which positions of the thief and the innocent suspect were interchanged, that is, the thief took position 5 and the innocent suspect position 3. Positions of the foils were randomized for each of the lineup versions. This resulted in a total of 12 versions of thief-present lineups, six versions of victim and six versions of bystander lineups.

A pilot study was conducted to establish effective lineup sizes. For each of the targets, we tested the target-absent lineup and one randomly selected version of the target-present lineup. Tredoux’s *E*s ranged from 3.5 to 4.6 (19 to 20 mock witnesses, total *N* = 119).

#### Procedure

The experiment was approved by the Ethics Review Committee Psychology and Neuroscience of Maastricht University. The experiment was performed in accordance with all the relevant guidelines and regulations. All participants provided informed consent to participate in the experiment. The data were collected between June and November. Participants who fulfilled the pre-screening criteria received access to the main study and a personal message inviting them for participation. As a cover story, we informed them that the experiment focused on the long-term effects of eating, caffeine-consumption habits and circadian rhythms on memory performance. We instructed participants not to consume alcohol for 8 h prior to testing, more than two cups of coffee on the day of testing and sleep a minimum of six hours in the night prior to testing to exclude the possibility of confounding effects on alertness and memory performance.

Testing sessions took place either between 7:30 a.m. and 9:00 a.m. or between 8:30 p.m. and 10:00 p.m. We used more extreme early morning and late evening hours compared to Experiment 1 to maximize the possible synchrony effect. Participants watched a stimulus film and were instructed to watch the film closely and pay attention to every detail and that they will be asked to act as eyewitnesses. Then, they provided answers to seven multiple choice questions concerning their food consumption habits (e.g., “How hungry are you usually in the morning?”); these were included for additional support of the cover story. Next, participants viewed the mug book. When all 16 mug shots had been presented, participants could make a selection or press the “Not present” option. As filler tasks, participants engaged in stem completion tasks adopted from Jacoby (1998) and an object search filler task for about 20 min.

Following the filler tasks, participants viewed the three lineups in succession and attempted to identify the thief, the victim and the bystander who were involved in the stimulus event. The thief lineup always appeared first. The remaining two lineups were presented in a random order. Prior to each identification, participants were informed which of the persons the lineup referred to and viewed a schematic of the crime scene with the silhouette of the respective character highlighted. They were informed that the targets may or may not be present in the lineups and were encouraged to select the “Not present” option if they were not sure or didn’t know. After each of the decisions, participants indicated their confidence. Decision times for each identification decision were recorded automatically.

At the end of the experiment, participants were presented with a block of data quality check items. The debriefing occurred upon completion of data collection.

### Results and discussion

#### Data analyses

The pre-registered analysis plan was based on the assumption that presenting a mug shot of an innocent suspect in the biased conditions would affect the further identification decision in lineups. Because this manipulation was unsuccessful, our analyses deviated from the pre-registered analysis plan in two respects. First, we could not analyze the effects of our predictors on innocent suspect misidentifications. Second, we did not include the factor bias in our analyses of identification accuracy in the perpetrator lineups (i.e., we treated all identification decisions as unbiased) and hence conducted one analysis across all lineup decisions.

We ran all analyses twice, once including the additionally collected 12 responses and once excluding them. The pattern of results was analogous. Therefore, we report statistical output for analyses performed including data collected from all participants.

We were unable to verify whether 10 participants adhered to the requirements of participating within the specified timeframes. We re-ran all the analyses excluding these participants and obtained analogous results. Nonetheless, we excluded data from these participants from analyses due to importance of time of day in our experimental design.

#### Identification performance

To test the effects of time-of-day optimality and target-presence on the likelihood that participants made an accurate identification decision, we entered time-of-day optimality, target-presence, and their two-way interaction in a GEE model. We deleted non-significant terms stepwise. The final model only included the main effect of target presence: participants had higher odds of making an accurate decision from target-absent compared to target-present lineups, Wald χ^2^(1) = 9.08, Exp(B) = 1.51, *p* = .002. The effect of testing optimality did not materialize. Frequencies and proportions of different lineup outcomes for each of the lineups can be found in Table 4.

**Table 4:** Frequencies and proportions of different lineup outcomes at optimal and non-optimal time of day in Experiment 2.

Lineup	Optimal		Non-optimal	
	Frequency	Proportion	Frequency	Proportion
**Thief**				
*Target-present*				
Correct identifications	19	21.3%	11	15.3%
Foil identifications	20	22.5%	15	20.8%
Incorrect rejections	50	56.2%	46	63.9%
Total	89	100%	72	100%
*Target-absent*				
Correct rejections	58	67.4%	44	64.7%
Foil identifications	28	32.6%	24	35.3%
Total	86	100%	68	100%
**Victim**				
*Target-present*				
Correct identifications	55	59.2%	41	61.2%
Foil identifications	19	20.4%	14	20.9%
Incorrect rejections	19	20.4%	12	17.9%
Total	93	100%	67	100%
*Target-absent*				
Correct rejections	51	62.2%	32	43.8%
Foil identifications	31	37.8%	41	56.2%
Total	82	100%	73	100%
**Bystander**				
*Target-present*				
Correct identifications	45	54.8%	41	56.0%
Foil identifications	19	23.2%	16	22.0%
Incorrect rejections	18	22.0%	16	22.0%
Total	82	100%	73	100%
*Target-absent*				
Correct rejections	39	41.9%	27	40.3%
Foil identifications	54	58.1%	40	59.7%
Total	93	100%	67	100%

Therefore, we did not replicate the unusual findings obtained in Experiment 1, where performance in target-present lineups was better at non-optimal compared to optimal time of day. We also did not observe the synchrony effect previously reported in recognition performance of non-facial stimuli^18,19^.

##### Differential effect of testing optimality in owls and larks

We ran exploratory analysis to test the possibility that the effect of testing optimality on identification accuracy manifested itself differently in participants with morning and evening chronotype. We entered chronotype (morningness versus eveningness), test time (morning vs evening), target-presence (target-present versus target-absent), their two-way and three-way interactions as predictors into the model. The final model included the main effect of target presence, Wald χ^2^(1) = 9.62, Exp(B) = 1.51, *p* = .002, and chronotype, Wald χ^2^(1) = 8.51, Exp(B) = 1.46, *p* = .004. This means that participants had higher odds of making an accurate decision from target-absent than target-present lineups and participants with evening preference were more likely to make and accurate identification decision than those with morning preference. The expected chronotype x test time interaction was not significant, indicating that the effect of chronotype occurred irrespective of the time of the day when testing occurred.

##### Sensitivity and response bias

Paralleling the exploratory signal detection analyses performed on data in Experiment 1, discriminability did not significantly differ between optimality conditions, with *d′*_Non-Opt_ = 1.18 versus *d′*_Opt_ = 1.41, *G* = 0.66, *p* = .94. The response bias measures were again comparable for the two conditions, *c*_Non-Opt_ = 0.76; *c*_Opt_ = 0.79.

**Figure 3:**
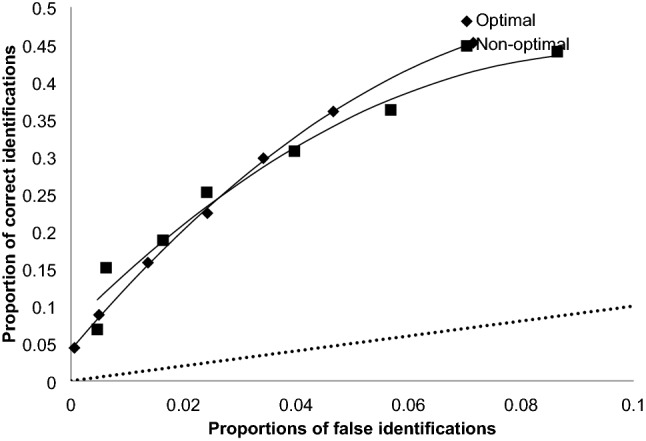
Receiver operating characteristics plots for optimal and non-optimal testing sessions across all lineups in Experiment 2.

Figure 3 displays the ROC curves for optimal versus non-optimal testing conditions, with the diagonal line representing chance performance. The two curves nearly overlap, confirming that there was no benefit from matching participant’s time-of-day preference at any of the confidence cut-off levels.

To summarize, we observed no benefit for eyewitness identification decisions of matching testing time to participants’ circadian preference. The finding that evening-type participants performed significantly better than morning-type participants may be attributable to differences in information processing styles between the two chronotypes. Evening time-of-day preference have been previously linked to superior performance in holistic information processing and appear to be better at processing non-verbal and emotional stimuli^56^, that is, factors that are known to be relevant for face processing^57,58^.

#### Effect of time-of-day optimality on the confidence-accuracy relationship

To test the effect of time-of-day optimality on the confidence-accuracy relationship, we entered identification accuracy (accurate versus inaccurate), choosing (selection versus rejection), and time-of-day optimality (optimal versus non-optimal), as well as their two- and three-way interactions as predictors in the initial GEE model. The final model contained a significant Accuracy × Choosing × Optimality interaction, Wald χ^2^(1) = 4.99, *p* = .026. Separate analyses split by time-of-day optimality revealed a significant simple Accuracy x Choosing interaction at non-optimal, Wald χ^2^(1) = 14.92, *p* < .001, but not optimal time of day, Wald χ^2^(1) = 0.65, *p* = .420. Further analyses performed on choosers and nonchoosers that were tested at non-optimal time of day showed that accurate choosers were more confident than inaccurate choosers, *b* = 1.36, Wald χ^2^(1) = 29.45, *p* < .001. This effect was not statistically significant for nonchoosers, *b* = 0.16, Wald χ^2^(1) = 0.69, *p* = .408. Contrary to the findings of Experiment 1, these results show that time-of-day optimality did affect the postdictive value of confidence. However, the direction of the effect was opposite than we hypothesized: The confidence-accuracy relationship in choosers was stronger at non-optimal compared to optimal time of day.

##### Calibration of confidence measures

The confidence-accuracy correlation was significant for choosers, *r*(527) = 0.27, *p* < .001, and for nonchoosers, *r*(414) = 0.106, *p* = .031. Figure 2 (panel C) displays the confidence-accuracy calibration curves for choosers at optimal (number of observations *n* = 300) and non-optimal (*n* = 250) time of day. Visual inspection of the curves reveals no significant differences between optimal and non-optimal testing (all the confidence intervals overlap). Similarly, nonchoosers did not show any significant differences between the two conditions: The calibration curves for both optimal (*n* = 240) and non-optimal (*n* = 182) sessions almost parallel the X-axis (see panel D of Fig. 2). In line with findings obtained in analogous explorative analyses in Experiment 1, the confidence intervals for all calibration statistics overlap (see Table 3).

#### Effect of time-of-day optimality on the decision time-accuracy relationship

To establish the effect of time-of-day optimality on the decision-time-accuracy relationship, we entered identification accuracy (accurate versus inaccurate), choosing (selection versus rejection), and time-of-day optimality (optimal versus non-optimal) in a GEE model. As in Study 1, we entered log-transformed decision times (log base 10) as the dependent variable due to significant positive skewness and kurtosis. The final model contained only the main effect of Choosing, *b* = 0.05, Wald χ^2^(1) = 10.99, *p* = .001. Participants who made a selection were faster than those who rejected the lineup.

These findings do not replicate the findings of Experiment 1, where non-optimal testing resulted in eradication of the predictive value of decision times on identification accuracy. We also did not replicate the commonly reported effect in which accurate choosers show faster decision times compared to inaccurate choosers^20–24^.

Our additional interest was in whether non-optimal time of day magnifies unwanted influence of factors that are known to have a biasing effect on eyewitness identifications. To explore this possibility, we exposed some of our participants to a mug shot of an innocent suspect prior to administering the lineup. This manipulation has shown to generate biased outcomes in subsequent identification procedures when a lineup includes the photograph of the aforementioned innocent suspect^44^. Our interest was whether non-optimal testing would increase this biasing effect. Unfortunately, the mug shot manipulation did not appear to be successful. One potential explanation for this could be the short retention interval between exposure to mug shots and the subsequent lineup. We used a retention interval of about 20 min, as opposed to a minimum of 48 h and up to a week in prior research^59–61^. The difficulties of conducting multi-session experiments in the MTurk environment did not allow for an experimental design with a longer retention interval. Future research employing a successful biasing manipulation can investigate the possibility that non-optimal testing may magnify the detrimental effect of the factors that are known to reduce identification accuracy.

## General discussion

Over the decades, research has collected sufficient evidence showing that that time of day can affect cognitive performance across an array of cognitive domains, including memory performance. This guided our interest in investigating whether time of day is also a factor of relevance for the eyewitness memory field, specifically for eyewitness identifications. We expected that testing participants at optimal as opposed to non-optimal time of day would have beneficial effect on identification accuracy. We also hypothesized that optimal testing would strengthen the postdictive value of confidence and decision times. To the best of our knowledge, this is the first attempt to investigate this effect in these contexts. Overall, results of the two studies we conducted show that previously reported findings on superior memory performance during circadian peaks do not translate into the eyewitness identification domain in the straightforward manner we anticipated.

In terms of identification accuracy, we found no support for the hypothesis that identification accuracy would be higher during circadian peaks as opposed to circadian troughs. These findings run contrary to the previous literature: Typically, reports recall and recognition of verbal and pictorial stimuli is superior at optimal time of day^17–19^. This discrepancy is likely to result from differences between the eyewitness memory paradigm we relied upon and methodology of prior experiments that report the synchrony effect patterns in recognition performance.

The specifics of retrieval instructions are perhaps among the most important of such differences. Consistent with standard methodologies in eyewitness memory research, identification instructions clearly specified that the perpetrator may or may not be present in the lineup. We also encouraged participants to select the “Not present” option if they were not sure or did not know. No equivalent of such instructions was present in the protocols of previous studies that showed synchrony effects in memory performance^11^. Such instructions are known to induce a more conservative response strategy, encouraging a neutral position towards the presence of the target in the lineup^62^.

Furthermore, we informed participants before presenting the stimulus event that they would be asked to serve as eyewitnesses. Efficient allocation of limited cognitive resources can effectively counteract the dropdown in attentional capacities during non-optimal hours of the day^63^. Prior knowledge on the nature of the memory test may have affected our participants’ encoding strategies, encouraging efficient distribution of attentional resources to enhance task-specific retrieval accuracy. In comparison to prior synchrony effect research, it is possible that our experimental protocols provided participants more opportunity to prioritize the allocation of their attentional capacities in non-optimal sessions, allowing them to offset the potential impairing effects of circadian troughs.

We would like to point out that the abovementioned methodological peculiarities serve the purpose of increasing ecological validity of eyewitness memory experiments compared to other types of recognition models. Real-life eyewitnesses may often (though not always) realize they are witnessing a crime; thus, their encoding strategies would significantly differ from those of participants who are studying word lists in a prototypical psychological lab. In a similar manner, the retrieval instructions we used mimic high standards of police practice, encouraging mock witnesses to take the recognition decision more seriously compared to, say, recognizing words, sentences or pictures in the lab. Therefore, the discrepancy of our findings with the previous literature may serve as an example of difficulties one may face when applying findings of basic laboratory studies to applied settings in an overly straightforward manner.

The present experiments are also the first attempt to test synchrony effects in face recognition performance. Could our findings indicate that face recognition is affected by circadian variations in arousal in a different manner than recognition of other types of stimuli? After all, face recognition is traditionally thought to rely on cognitive processes that are relatively independent from other types of recognition memory^64^. Processing of faces also differs from processing of non-facial stimuli in terms of allocation of attention^65–67^. However, we would refrain from generalizing our findings to face recognition performance in general. Even though lineup identification procedures are a subtype of recognition tests, they differ from traditional recognition tasks in numerous ways (e.g., exposure duration, the presence of fillers, conservative identification instructions etc.) Therefore, future research employing traditional face recognition paradigm with a significantly higher number of recognition trials is necessary to more thoroughly investigate the circadian variations in face recognition performance.

Our second focus of interest was whether testing optimality would affect the relationship between identification accuracy and its postdictors. Based on the optimality hypothesis^25^, we expected a stronger confidence-accuracy relationship in choosers at optimal compared to non-optimal time of day. Results in Experiment 1 showed that confidence-accuracy relationship was not moderated by testing optimality, whereas in Experiment 2, the postdictive value of confidence was in fact stronger at non-optimal compared to optimal time of day. In addition, participants in Experiment 1 were significantly better calibrated in some of the confidence categories at non-optimal compared to optimal time of day. Combined, these findings suggest that circadian effects on confidence-accuracy relationship may be better explained by a competing theory-driven confidence judgments hypothesis^68^. This hypothesis states that, if aware of the factor that negatively affects memory, participants may take this information into account and adjust their metamemory judgements appropriately. This might have been the case in the current experiments; that is, our participants may have been aware of the possible cognitive impairments due to the circadian troughs and taken this information into account when indicating their post-decision confidence.

Testing optimality strengthened the decision time-accuracy in Experiment 1, as expected, but not Experiment 2. We should note that Experiment 2 also did not replicate the finding that accurate choosers make faster identification decisions than nonchoosers^20–24^. This might be the result of reduced control over the experimental environment in Experiment 2, which was conducted online, as opposed to Experiment 1, which was conducted in laboratory settings. If replicated in future studies, the findings of Experiment 1 would be interesting in terms of the predictive value of decision times in eyewitness identification decisions. They may also be of interest to memory researchers who rely on decision times as their outcome measure: it may be important to take into account that time of the day when the test is administered may confound the decision-time-based outcomes.

It is important to note that our findings are limited to situations when all other factors that are known to affect memory performance are optimal. In both of the experiments conducted, the stimulus film presentation occurred at the beginning of the testing session, eliminating possible fatigue effects. Participants were asked to watch the stimulus film closely and warned that they would later be asked to act as eyewitnesses. Each of the targets’ faces was visible for a sufficient amount of time to ensure robust encoding strength^69^. All identification instructions were consistent with good practice in the eyewitness identification procedures^62^. In real-life scenarios, however, encoding conditions are not always optimal, nor are the identification procedures always best examples of good police practices. Additionally, delays between witnessing the event and the memory test in applied settings are typically longer compared with retention intervals in our experiments. Finally, certain categories of eyewitnesses may be especially prone to memory errors; for instance, this concerns the elderly participants due to the age decline in memory performance^70^. It remains unclear whether non-optimal testing would have an additive detrimental effect under such conditions. Future research in this direction is necessary. In addition, future research may use more advanced self-report tools to determine participants’ time-of-day preference, such as the Munich Chronotype Questionnaire^71^.

Creating optimal retrieval conditions for eyewitnesses is important for reducing identification errors and the associated potential of miscarriages of justice. This necessitates a close examination of factors that contribute to memory performance in eyewitnesses. We tested whether time-of-day optimality could be another factor of high relevance to the eyewitness memory field. For instance, is it possible to increase identification accuracy of eyewitnesses by administering the lineup at peak hour of the day? Based on our results, the takeaway message for the policymakers is straightforward: we found no evidence supporting the idea that daily variations in performance affect eyewitness identification performance. In other words, with respect to time-of-day optimality, neither the time at which the event was witnessed nor the timing of the lineup administration appears to affect identification outcome.

## References

[1] Hastings MH, Reddy AB, Maywood ES (2003). A clockwork web: Circadian timing in brain and periphery, in health and disease. Nat. Rev. Neurosci..

[2] Smolensky MH, Peppas NA (2007). Chronobiology, drug delivery, and chronotherapeutics. Adv. Drug. Deliv. Rev..

[3] Peek CB (2017). Circadian clock interaction with HIF1alpha mediates oxygenic metabolism and anaerobic glycolysis in skeletal muscle. Cell Metab..

[4] Kelley P, Lockley SW, Foster RG, Kelley J (2014). Synchronizing education to adolescent biology: ‘Let teens sleep, start school later’. Learn. Media Technol..

[5] Clark SE, Benjamin AS, Wixted JT, Mickes L, Gronlund SD (2015). Eyewitness identification and the accuracy of the criminal justice system. Policy Insights Behav. Brain Sci..

[6] Halberg F (2003). Transdisciplinary unifying implications of circadian findings in the 1950s. J. Circadian Rhythms.

[7] Czeisler CA, Gooley JJ (2007). Sleep and circadian rhythms in humans. Cold Spring Harb. Symp. Quant. Biol..

[8] Horne JA, Ostberg O (1976). A self-assessment questionnaire to determine morningness-eveningness in human circadian rhythms. Int. J. Chronobiol..

[9] Levandovski R, Sasso E, Hidalgo MP (2013). Chronotype: A review of the advances, limits and applicability of the main instruments used in the literature to assess human phenotype. Trends Psychiatry Psychother..

[10] Adan A (2012). Circadian typology: A comprehensive review. Chronobiol. Int..

[11] Schmidt C, Collette F, Cajochen C, Peigneux P (2007). A time to think: Circadian rhythms in human cognition. Cogn. Neuropsychol..

[12] May CP (1999). Synchrony effects in cognition: The costs and a benefit. Psychon. Bull. Rev..

[13] May CP, Hasher L (1998). Synchrony effects in inhibitory control over thought and action. J. Exp. Psychol. Hum. Percept. Perform..

[14] Bodenhausen GV (1990). Stereotypes as judgmental heuristics: Evidence of circadian variations in discrimination. Psychol. Sci..

[15] May CP, Hasher L, Foong N (2005). Implicit memory, age, and time of day: Paradoxical priming effects. Psychol. Sci..

[16] Anderson MJ, Petros TV, Beckwith BE, Mitchell WW, Fritz S (1991). Individual differences in the effect of time of day on long-term memory access. Am. J. Psychol..

[17] Petros TV, Beckwith BE, Anderson M (1990). Individual differences in the effects of time of day and passage difficulty on prose memory in adults. Br. J. Psychol..

[18] May CP, Hasher L, Stoltzfus ER (1993). Optimal time of day and the magnitude of age differences in memory. Psychol. Sci..

[19] Intons-Peterson MJ, Rocchi P, West T, McLellan K, Hackney A (1999). Age, testing at preferred or nonpreferred times (testing optimality), and false memory. J. Exp. Psychol. Learn. Mem. Cogn..

[20] Brewer N, Wells GL (2006). The confidence-accuracy relationship in eyewitness identification: Effects of lineup instructions, foil similarity, and target-absent base rates. J. Exp. Psychol. Appl..

[21] Dunning D, Stern LB (1994). Distinguishing accurate from inaccurate eyewitness identifications via inquiries about decision processes. J. Pers. Soc. Psychol..

[22] Sauerland M, Sporer SL (2007). Post-decision confidence, decision time, and self-reported decision processes as postdictors of identification accuracy. Psychol. Crime Law.

[23] Sauerland M, Sporer SL (2009). Fast and confident: Postdicting eyewitness identification accuracy in a field study. J. Exp. Psychol. Appl..

[24] Sporer SL, Penrod S, Read D, Cutler B (1995). Choosing, confidence, and accuracy: A meta-analysis of the confidence-accuracy relation in eyewitness identification studies. Psychol. Bull..

[25] Deffenbacher KA (1980). Eyewitness accuracy and confidence: Can we infer anything about their relationship?. Law Hum. Behav..

[26] Sauer J, Hope L (2016). The effects of divided attention at study and reporting procedure on regulation and monitoring for episodic recall. Acta Psychol. (Amst).

[27] Adan A, Almirall H (1991). Horne & Östberg morningness-eveningness questionnaire: A reduced scale. Pers. Individ. Dif..

[28] Meissner CA, Brigham JC (2001). Thirty years of investigating the own-race bias in memory for faces: A meta-analytic review. Psychol. Pub. Pol'y & L..

[29] Wilson, J. P., Bernstein, M. J. & Hugenberg, K. A synthetic perspective on the own-race bias in eyewitness identification. In *Advances in Psychology and Law Vol. 2* (eds Bornstein, B.H. & Miller, M.K.) 241–270 (Springer International Publishing AG, New York, 2016).

[30] Mansour J, Beaudry J, Lindsay R (2017). Are multiple-trial experiments appropriate for eyewitness identification studies? Accuracy, choosing, and confidence across trials. Behav. Res. Methods.

[31] Di Milia L, Adan A, Natale V, Randler C (2013). Reviewing the psychometric properties of contemporary circadian typology measures. Chronobiol. Int..

[32] Sauerland M, Krix AC, van Kan N, Glunz S, Sak A (2014). Speaking is silver, writing is golden? The role of cognitive and social factors in written versus spoken witness accounts. Mem. Cognit..

[33] Brackmann N, Sauerland M, Otgaar H (2019). Developmental trends in lineup performance: Adolescents are more prone to innocent bystander misidentifications than children and adults. Mem. Cognit..

[34] Tredoux C (1999). Statistical considerations when determining measures of lineup size and lineup bias. Appl. Cogn. Psychol..

[35] Meissner CA, Brigham JC (2001). A meta-analysis of the verbal overshadowing effect in face identification. Appl. Cogn. Psychol..

[36] Buysse DJ, Reynolds CF, Monk TH, Berman SR, Kupfer DJ (1989). The Pittsburgh sleep quality index: A new instrument for psychiatric practice and research. Psychiatry Res..

[37] Zeger SL, Liang K-Y, Albert PS (1988). Models for longitudinal data: A generalized estimating equation approach. Biometrics.

[38] Chen H, Cohen P, Chen S (2010). How big is a big odds ratio? interpreting the magnitudes of odds ratios in epidemiological studies. Commun. Stat. Simul. Comput..

[39] Green D, Swets J (2000). Signal detection theory and psychophysics.

[40] Mickes L, Flowe HD, Wixted JT (2012). Receiver operating characteristic analysis of eyewitness memory: Comparing the diagnostic accuracy of simultaneous versus sequential lineups. J. Exp. Psychol. Appl..

[41] Weber N, Brewer N (2004). Confidence-accuracy calibration in absolute and relative face recognition judgments. J. Exp. Psychol. Appl..

[42] Flowe HD (2017). The Effects of alcohol intoxication on accuracy and the confidence-accuracy relationship in photographic simultaneous line-ups. Appl. Cogn. Psychol..

[43] Wixted JT, Mickes L (2014). A signal-detection-based diagnostic-feature-detection model of eyewitness identification. Psychol. Rev..

[44] Deffenbacher KA, Bornstein BH, Penrod SD (2006). Mugshot exposure effects: Retroactive interference, mugshot commitment, source confusion, and unconscious transference. Law Hum. Behav..

[45] Faul F, Erdfelder E, Lang AG, Buchner A (2007). G*Power 3: A flexible statistical power analysis program for the social, behavioral, and biomedical sciences. Behav. Res. Methods.

[46] Faul F, Erdfelder E, Buchner A, Lang AG (2009). Statistical power analyses using G*Power 3.1: Tests for correlation and regression analyses. Behav. Res. Methods.

[47] Mason W, Suri S (2012). Conducting behavioral research on Amazon's Mechanical Turk. Behav. Res. Methods.

[48] Paolacci G, Chandler J, Ipeirotis PG (2010). Running experiments on Amazon Mechanical Turk. Judgm. Decis. Mak..

[49] Buhrmester M, Kwang T, Gosling SD (2011). Amazon's Mechanical Turk: A new source of inexpensive, yet high-quality, data?. Perspect. Psychol. Sci..

[50] Crump MJ, McDonnell JV, Gureckis TM (2013). Evaluating Amazon's Mechanical Turk as a tool for experimental behavioral research. PLoS ONE.

[51] Rand DG (2012). The promise of Mechanical Turk: How online labor markets can help theorists run behavioral experiments. J. Theor. Biol..

[52] Meade AW, Craig SB (2012). Identifying careless responses in survey data. Psychol. Methods.

[53] Fleischer A, Mead AD, Huang J (2015). Inattentive responding in mturk and other online samples. Ind. Organ. Psychol..

[54] Oppenheimer DM, Meyvis T, Davidenko N (2009). Instructional manipulation checks: Detecting satisficing to increase statistical power. J Exp. Soc. Psychol..

[55] Sharpe Wessling K (2017). MTurk character misrepresentation: Assessment and solutions. J. Consum. Res..

[56] Fabbri M, Antonietti A, Giorgetti M, Tonetti L, Natale V (2007). Circadian typology and style of thinking differences. Learn. Individ. Differ..

[57] Haxby JV, Hoffman EA, Gobbini MI (2002). Human neural systems for face recognition and social communication. Biol. Psychiatry.

[58] Tanaka JW, Farah MJ (1993). Parts and wholes in face recognition. Q. J. Exp. Psychol. A.

[59] Goodsell CA, Neuschatz JS, Gronlund SD (2009). Effects of mugshot commitment on lineup performance in young and older adults. Appl. Cogn. Psychol..

[60] Memon A, Hope L, Bartlett J, Bull R (2002). Eyewitness recognition errors: The effects of mugshot viewing and choosing in young and old adults. Mem. Cognit..

[61] Perfect TJ, Harris LJ (2003). Adult age differences in unconscious transference: Source confusion or identity blending?. Mem. Cognit..

[62] Steblay NM (1997). Social influence in eyewitness recall: A meta-analytic review of lineup instruction effects. Law Hum. Behav..

[63] Nowack K, Van Der Meer E (2018). The synchrony effect revisited: Chronotype, time of day and cognitive performance in a semantic analogy task. Chronobiol. Int..

[64] Robotham RJ, Starrfelt R (2017). Face and word recognition can be selectively affected by brain injury or developmental disorders. Front. Psychol..

[65] Bindemann M, Burton AM, Jenkins R (2005). Capacity limits for face processing. Cognition.

[66] Boutet I, Chaudhuri A (2001). Multistability of overlapped face stimuli is dependent upon orientation. Perception.

[67] Palermo R, Rhodes G (2002). The influence of divided attention on holistic face perception. Cognition.

[68] Palmer MA, Brewer N, Weber N, Nagesh A (2013). The confidence-accuracy relationship for eyewitness identification decisions: Effects of exposure duration, retention interval, and divided attention. J. Exp. Psychol. Appl..

[69] Memon A, Hope L, Bull R (2003). Exposure duration: Effects on eyewitness accuracy and confidence. Br. J. Psychol..

[70] Fitzgerald RJ, Price HL (2015). Eyewitness identification across the life span: A meta-analysis of age differences. Psychol. Bull..

[71] Roenneberg T, Wirz-Justice A, Merrow M (2003). Life between clocks: Daily temporal patterns of human chronotypes. J. Biol. Rhythms.

